# Glucocorticoid-dependent transcription in skin requires epidermal expression of the glucocorticoid receptor and is modulated by the mineralocorticoid receptor

**DOI:** 10.1038/s41598-020-75853-5

**Published:** 2020-11-03

**Authors:** Lisa M. Sevilla, Judit Bigas, Álvaro Chiner-Oms, Iñaki Comas, Vicente Sentandreu, Paloma Pérez

**Affiliations:** 1 Instituto de Biomedicina de Valencia-Consejo Superior de Investigaciones Científicas (IBV-CSIC), Jaime Roig 11, 46010 Valencia, Spain; 2 Universidad de Valencia, Valencia, Spain

**Keywords:** Endocrinology, Hormones, Diseases, Skin diseases

## Abstract

Glucocorticoid (GC) actions are mediated through two closely related ligand-dependent transcription factors, the GC receptor (GR) and the mineralocorticoid receptor (MR). Given the wide and effective use of GCs to combat skin inflammatory diseases, it is important to understand the relative contribution of these receptors to the transcriptional response to topical GCs. We evaluated the gene expression profiles in the skin of mice with epidermal-specific loss of GR (GR^EKO^), MR (MR^EKO^), or both (double KO; DKO) in response to dexamethasone (Dex). The overall transcriptional response was abolished in GR^EKO^ and DKO skin suggesting dependence of the underlying dermis on the presence of epidermal GR. Indeed, the observed dermal GC resistance correlated with a constitutive decrease in GR activity and up-regulation of p38 activity in this skin compartment. Upon Dex treatment, more than 90% of differentially expressed genes (DEGs) in CO overlapped with MR^EKO^. However, the number of DEGs was fourfold increased and the magnitude of response was higher in MR^EKO^ vs CO, affecting both gene induction and repression. Taken together our data reveal that, in the cutaneous transcriptional response to GCs mediated through endogenous receptors, epidermal GR is mandatory while epidermal MR acts as a chief modulator of gene expression.

## Introduction

Glucocorticoid (GC) derivatives are widely used as topical treatments to combat skin inflammatory diseases due to their anti-proliferative and anti-inflammatory actions^1–3^. However, the outcome of GC treatments is highly variable among patients, with differences in sensitivity occurring due to a variety of factors, many of which are still to be defined. GC response can be influenced by heritable mutations or polymorphisms in GR and resistance that develops as a consequence of pathological processes^4^. Some patients show adverse effects following short treatments while others tolerate GCs well for longer time periods^4^. Inadequate GC therapy, due to generalized rather than patient-specific treatment strategies, results in reduced efficacy and also constitutes a socio-economic burden with considerable impact on health care costs. Moreover, detrimental side effects ranging from skin thinning to impaired wound healing preclude long term treatments^5^. Understanding differential responses among patients holds promise to optimize current GC based therapies minimizing unwanted effects and maximizing therapeutic responses.

GC effects can be mediated through the closely related GC receptor (GR) and the mineralocorticoid receptor (MR), which are ligand-dependent transcription factors^3^. We previously assessed the relative roles of these receptors in the skin by generating mice with epidermal-specific inactivation of GR, MR, or both (hereafter, GR epidermal KO/GR^EKO^, MR epidermal KO/MR^EKO^, or double GR/MR epidermal KO/DKO, respectively)^6–8^. The characterization of these mouse models demonstrated that: i) during development, epidermal GR and MR play non-overlapping functions and act cooperatively to regulate skin morphogenesis; and ii) in adulthood, neither GR nor MR are required in the epidermis under basal circumstances; however, both act as anti-inflammatory mediators in pathological conditions.

We have also demonstrated that both epidermal GR and MR are required for the anti-proliferative and protective actions of GCs in inflamed skin as the effects of Dex were drastically reduced in GR^EKO^, MR^EKO^, and DKO mice^8^. However, while GC targets have been previously reported in human cultured keratinocytes and skin^9,10^, the relative contribution of GR and MR to GC actions remains unknown. This study aims to understand the global contribution of the endogenous GC receptors, individual or combined, to the GC transcriptional response in the whole tissue, by using an in vivo approach.

## Results

To understand the relative contribution of epidermal GR and MR to the skin transcriptional response to GCs, we topically treated CO, GR^EKO^, MR^EKO^, and DKO mice^6–8^ with dexamethasone (Dex) or vehicle (V) for 24 h. RNA-sequencing was performed using whole skin and differentially expressed genes (DEGs) within each genotype were identified by calculating the fold-change (FC) of Dex vs V (Dataset S1; FDR < 0.05).

**Figure 1:**
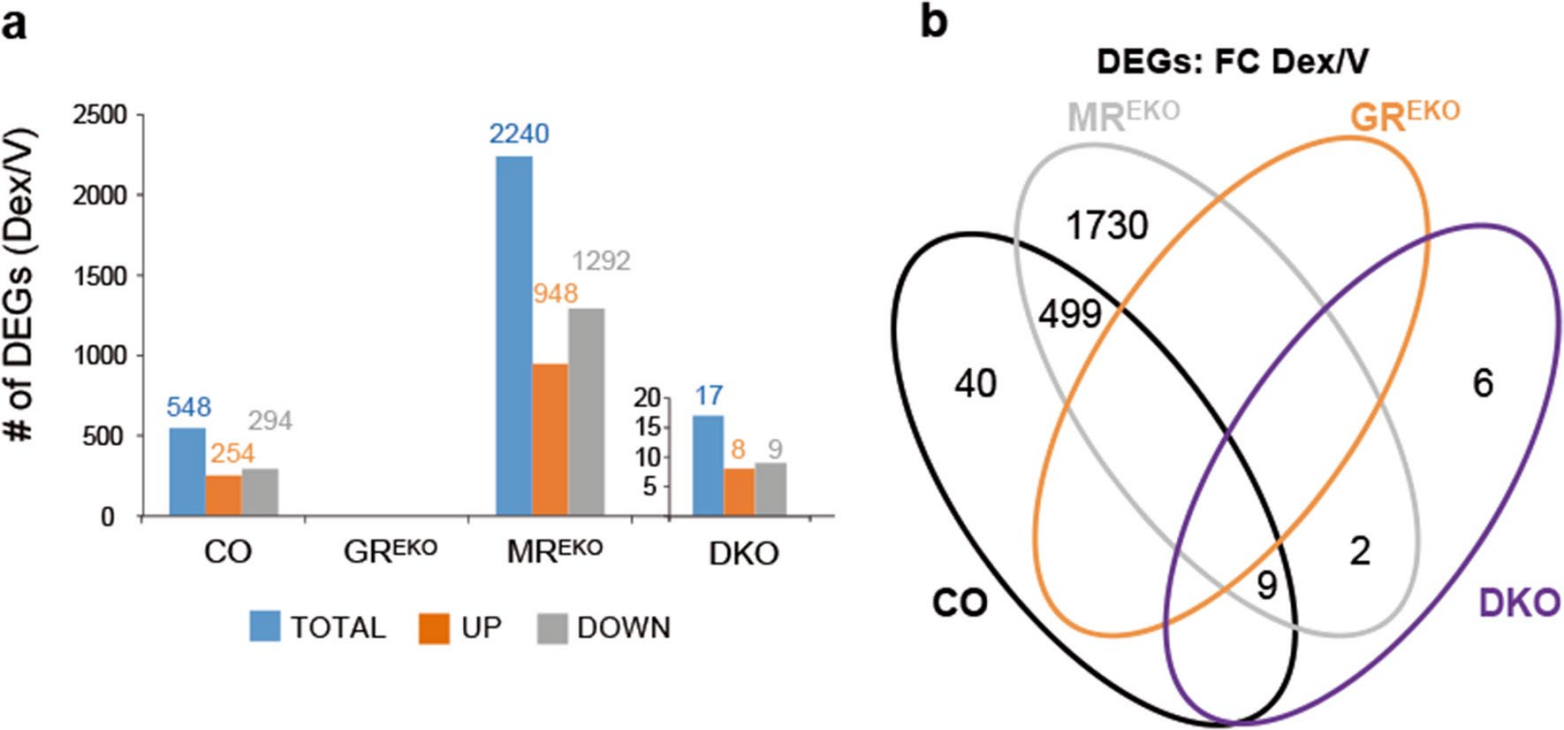
Response to topical glucocorticoids in CO, GR^EKO^, MR^EKO^, or DKO mouse skin. (**a**) Differentially Expressed Genes (DEGs) in CO, GR^EKO^, MR^EKO^, or DKO skin, topically treated with Dex or vehicle (V) for 24 h. DEGs in DKO are shown as an inset. Note the dramatic loss of transcriptional response in GR^EKO^ and DKO skin and the increase in DEGs in MR^EKO^. (**b**) Venn diagrams comparing lists of DEGs. Note the majority of DEGs identified in CO overlap with those identified in MR^EKO^.

Dex treatment affected the expression of 548 genes in CO skin (Fig. 1a; 254 genes up-regulated, 294 down-regulated). Importantly, GC-dependent transcription was totally abolished in GR^EKO^ skin while the loss of epidermal MR had a major impact on the GC response as 2240 genes were regulated by Dex in MR^EKO^ skin, a fourfold increase relative to CO (Fig. 1a; 948 genes up-regulated, 1292 down-regulated). The overlapping of DEGs between CO and MR^EKO^ was more than 90% (Fig. 1b). The response to GCs in DKO skin was virtually abolished with only 17 DEGs being identified (Fig. 1a; 8 up-regulated, 9 down-regulated).

It is feasible that the epidermal-specific loss of GR or MR induced changes in the expression of either receptor in the epidermal and/or dermal compartments as a mechanism of functional compensation. To address this, we assessed the basal expression of *Nr3c1* (GR) or *Nr3c2* (MR) in vehicle-treated CO, GR^EKO^, and MR^EKO^ mice, by RT-qPCR (Fig. S1). In CO mice, relative *Nr3c1* expression was higher in the epidermis as compared to the dermis while that of *Nr3c2* showed the opposite pattern, with higher levels in the dermis (Fig. S1; 0.5-fold and 3–4-fold, respectively). As expected, N*r3c1* expression was nearly absent in GR^EKO^ epidermis while N*r3c2* expression was undetectable in MR^EKO^ epidermis (Fig. S1). No other changes in N*r3c1/*N*r3c12* expression were detected between genotypes or compartments (Fig. S1).

### Epidermal GR is mandatory for the transcriptional response to GCs in skin

We have previously shown that the lack of GR in cultured keratinocytes almost completely eliminates the GC transcriptional response^7,11,12^, indicating that MR cannot compensate for its loss. However, what was most striking here was the lack of responsiveness to GCs in the whole skin of GR^EKO^ and DKO mice, suggesting dependence of the dermal compartment on the presence of epidermal GR. To elucidate this, CO and GR^EKO^ mice were topically treated with Dex or vehicle for 24 h, and after the separation of epidermis and dermis, the expression of the GC targets *Ddit4*, *Cebpd*, and *Fkbp51* was assessed by RT-QPCR in each genotype and compartment.

**Figure 2:**
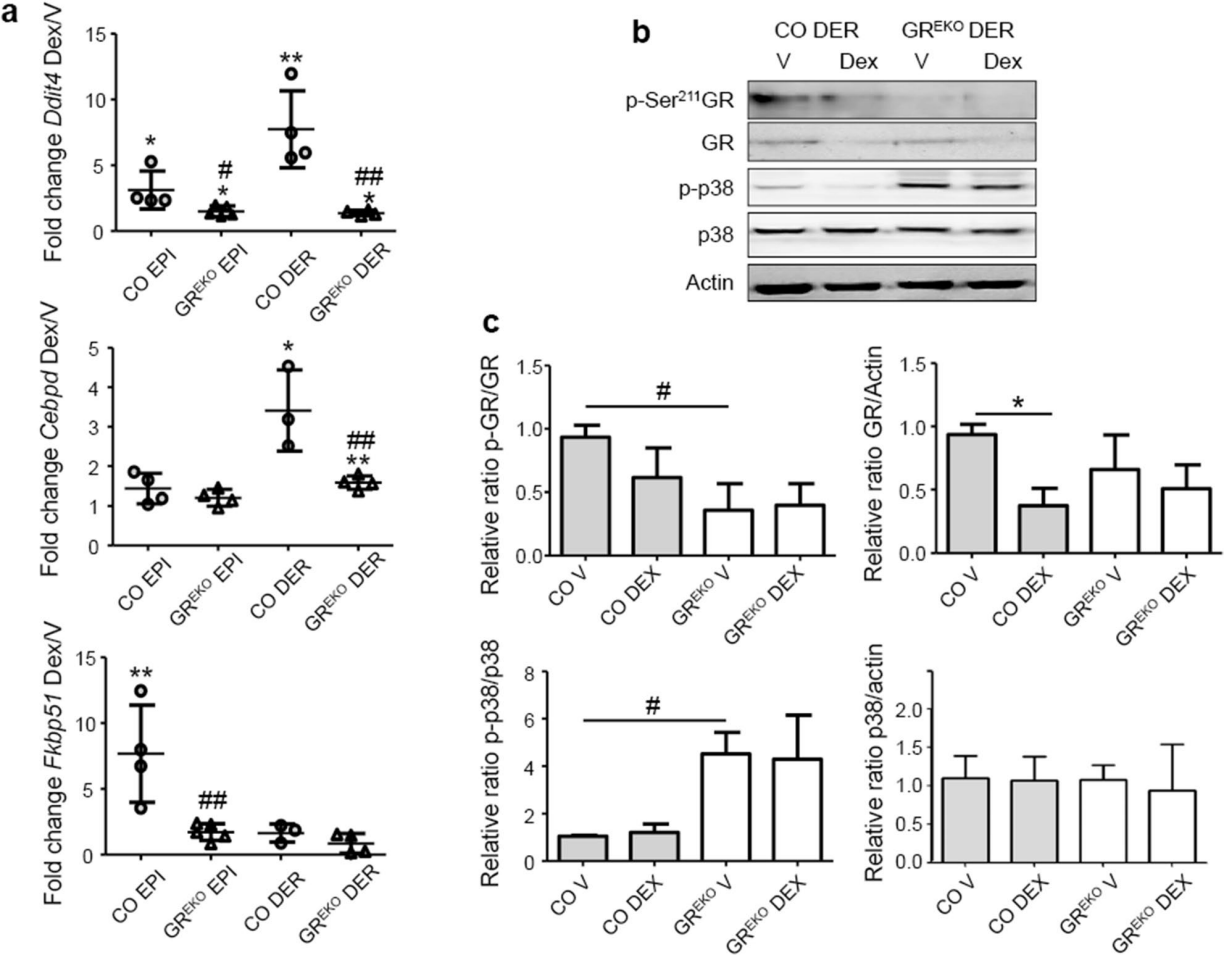
Glucocorticoid-dependent transcription is strongly impaired in GR^EKO^ epidermis and dermis. (**a**) Fold-change (FC) in expression of *Ddit4*, *Cebpd*, and *Fkbp51* in Dex *vs* Vehicle treated epidermis (EPI) or dermis (DER) was assessed by RT-qPCR in CO and GR^EKO^. n = 4 replicates per genotype and treatment. (**b**) Immunoblotting of CO and GR^EKO^ dermal extracts using specific antibodies for GR, p-GR (S211), p38, and p-p38. Actin was used as a loading control. Cropped blots are displayed; uncropped blots are included as Supplementary Information file S1. (**c**) Quantitation of protein expression. n = 3–5 replicates per genotype and treatment. In (**a**, **c**) statistically significant changes between Dex/V are denoted by asterisks and significant differences between genotypes by pound signs (*, ^#^p < 0.05; **, ^##^p < 0.01).

In the epidermis, Dex induced *Ddit4* and *Fkbp51* expression in CO, which was abolished in GR^EKO^; while *Cebpd* was not significantly up-regulated in the epidermis of either genotype (Fig. 2a). Importantly, the up-regulation of *Ddit4* and *Cebpd* in the dermis of Dex-treated CO skin was also strongly decreased in GR^EKO^; *Fkbp51* was not significantly induced in the dermis of either genotype (Fig. 2a). This decreased response to GCs correlated with basal decrease of GR activity (p-Ser 211/GR ratio) in dermal extracts from GR^EKO^ *vs* CO mice, as shown by immunoblotting (Fig. 2b,c; vehicle-treated samples). Also, the Dex-induced GR autologous down-regulation in dermal extracts from CO indicated an abnormal response to GCs in GR^EKO^ dermis (Fig. 2b,c).

We examined whether this GC-resistance was related to the constitutive activation of p38 MAPK, as reported for other cell types^13–20^. Immunoblotting followed by quantitation of p-p38/p38 ratio showed constitutive increase of p38 activity in dermal extracts of GR^EKO^ relative to CO (Fig. 2b,c; fourfold). These data indicate that the absence of epidermal GR can trigger GC resistance in the underlying dermis.

### Epidermal MR acts as a chief modulator of GR-dependent regulation

**Figure 3:**
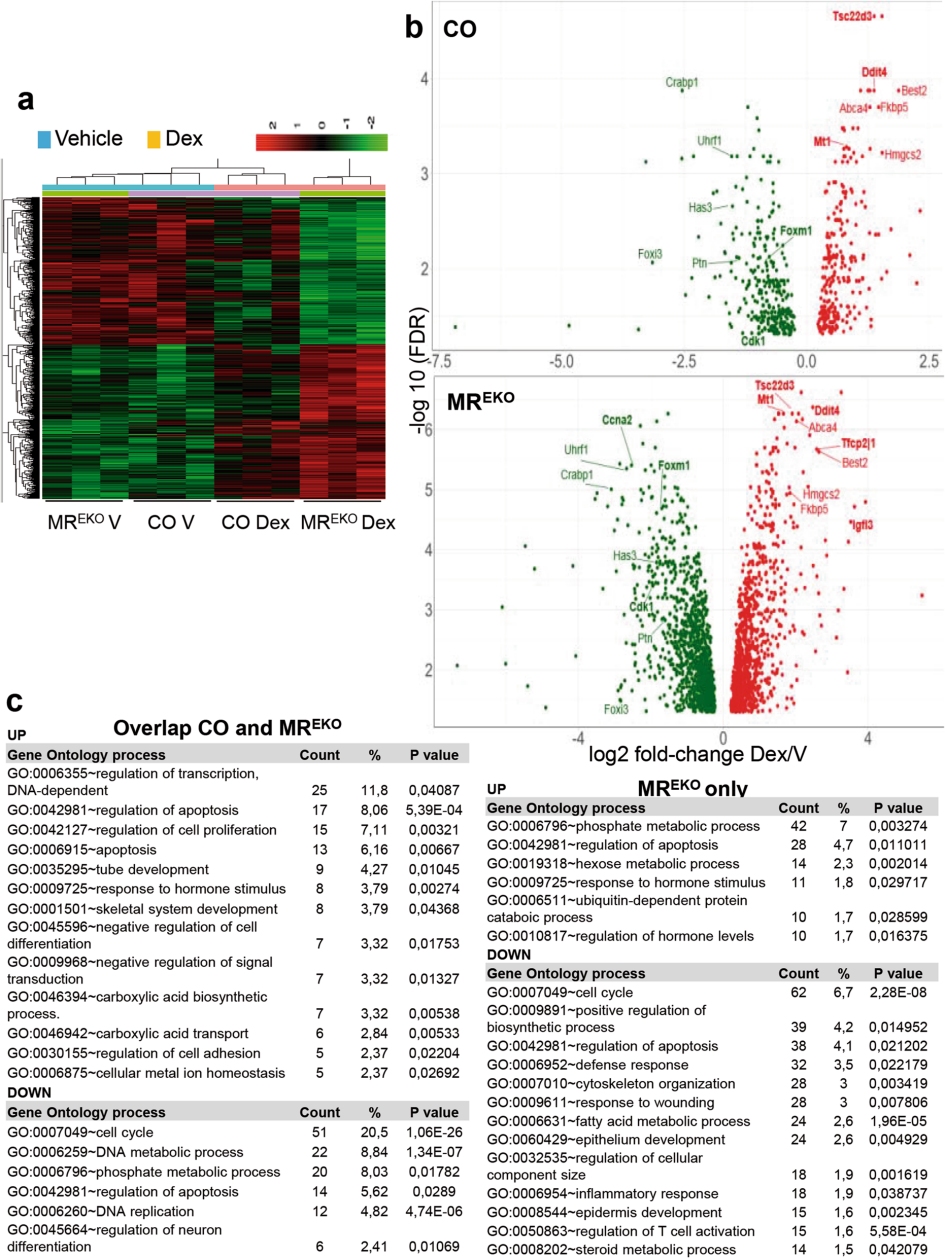
Heatmap and gene ontology clustering of Differentially Expressed Genes (DEGs) in CO and MR^EKO^ skin. (**a**) Heatmap of Differentially Expressed Genes (DEGs) in CO and MR^EKO^ skin, per genotype and treatment. Note that samples first cluster by treatment and then by genotype. The heat map was generated using the 'pheatmap' package (v 1.0.12) in the R statistical language (v 3.4; https://CRAN.R-project.org/package=pheatmap). (**b**) Log2 fold-change Dex/V *vs* Log10 adj-pvalue Dex/V. (**c**) Gene ontology (GO) clustering of DEGs that overlapped in CO and MR^EKO^ skin (top); or were uniquely identified in MR^EKO^ (bottom). The GO process, total count and percentage of genes, and p value, are indicated.

The increased number of genes regulated by Dex in MR^EKO^ vs CO skin pointed to epidermal MR as a chief modulator of the cutaneous response to GCs. A hierarchical clustering showed that in vehicle-treated samples the relative expression of DEGs was almost identical in CO and MR^EKO^ (Fig. 3a). In response to Dex, the pattern of gene expression changes was also similar in both genotypes (Fig. 3a). However, the overall magnitude of the response to Dex was more pronounced in MR^EKO^ relative to CO (Fig. 3a,b; note the scale of X axes). While the number of DEGs was fourfold higher in MR^EKO^ relative to CO, the proportion of up- and down-regulated genes was similar in both genotypes (Fig. S2, approximately 50%). Importantly, 95% of up-regulated genes and 90% of down-regulated genes in CO were coincident with those in MR^EKO^ (Fig. 3c).

Gene ontology (GO) clustering of DEGs identified in CO and MR^EKO^ revealed biological processes that were overrepresented in response to Dex regardless of the presence or absence of epidermal MR (Fig. 3c and Dataset S2). For the up-regulated genes, the more overrepresented category was regulation of transcription (11.8%), which highlights the relevance of short-term GC treatments on transcription factor expression. This category included well known GC-target genes such as *Zbtb16*, *Tsc22d3/Gilz1*, *Per1*, *Bcl6*, *Nfil3*, *Txnip*, and *Cebpd* (Dataset S2). Other enriched processes were regulation of apoptosis (11.8%) and cell proliferation (7.1%), including *Bcl2l1*, *Angptl4*, *Zbtb16*, and *Nfkbia* genes, among others. The highest percentage of genes down-regulated in both CO and MR^EKO^ were related to cell cycle (20.5%), including *E2f1*, *Aurka*, *Ccna2*, *Ccnd1*, *Uhrf1*, *Mki67*, *Cenpf*, *Birc5*, and *Plk1* (Fig. 3c and Dataset S2). Other overrepresented categories were DNA metabolic process (8.8%) and phosphate metabolic process (8%). Altogether, these data are consistent with the key role of GCs in the homeostasis of cutaneous tissue^2^.

GO clustering of the DEGs that appeared uniquely in the MR^EKO^ list identified several categories that were coincident to those overrepresented in gene sets common to CO and MR^EKO^, including regulation of apoptosis (4.6%; e.g. *Foxo1*, *Foxo3*, *Cited2*, *Vdr*, *Myc*, *Bcl2l11*, and *Cdkn1a*) and cell cycle (6.7%; e.g. *Aurkb*, *Ccne1*, *Ccnf*, *Cdk6*, *Cdk4*, *Chek1*, *Ccnb1*, and *Ccnb2*) (Fig. 3c and Dataset S2). These findings show that correct gene regulation by GCs occurs via MR-independent and -dependent manners.

Importantly, DEGs unique to MR^EKO^ also clustered into novel GO categories (Fig. 3c and Dataset S2). The up-regulated genes were related to phosphate metabolic process (7%), hexose metabolic process (2.3%), ubiquitin-dependent protein catabolic process (1.7%), and regulation of hormone levels (1.7%). Interestingly the genes uniquely down-regulated in MR^EKO^ clustered into categories that were crucial for GC-mediated responses in the cutaneous tissue such as defense response (3.4%), cytoskeleton organization (3%), response to wounding (3%), fatty acid metabolism (2.6%), epithelium development (2.6%), inflammatory response (1.9%), regulation of T cell activation (1.7%), and steroid metabolic process (1.5%) (Fig. 3c and Dataset S2). These categories included key genes such as *Cxcl9*, *Cxcl10*, *Tnf*, *Ptgs2*, *Krt14*, *Edaradd*, *Fdps*, and *Sc5d*.

**Figure 4:**
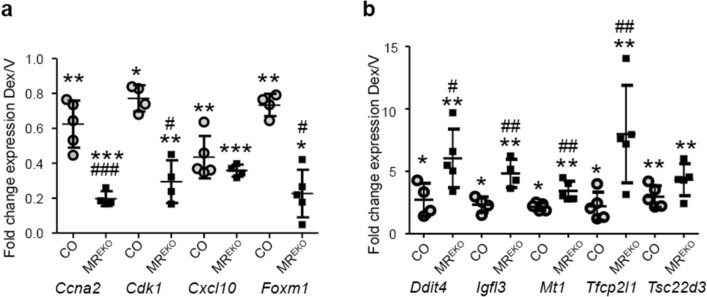
The loss of epidermal MR has a major impact in the cutaneous response to topical glucocorticoids. Fold change of indicated genes (Dex/V) assessed in control (CO) and MR^EKO^ skin by RT-qPCR. (**a**) Down-regulated genes. (**b**) Up-regulated genes. Statistically significant changes between Dex/V within each genotype are denoted by asterisks and significant differences between fold changes in CO and MR^EKO^ by pound signs (*, ^#^p < 0.05; **, ^##^p < 0.01; ***, ^###^p < 0.001).

Gene expression changes were confirmed by RT-qPCR. Cell cycle related genes such as *Ccna2*, *Cdk1*, and *Foxm1*, and the chemokine *Cxcl10* were repressed by Dex while *Ddit4*, *Igfl3*, *Mt1*, *Tfcp2l1*, and *Tsc22d3/Gilz1*, were induced (Fig. 4). Importantly, all the genes assessed showed significant differences in the magnitude of response of CO and MR^EKO^, except for *Cxcl10* and *Tsc22d3/Gilz1* (Fig. 4). These results indicate that the lack of epidermal MR results in more pronounced transcriptional response to Dex in skin, affecting both gene induction and repression.

**Figure 5:**
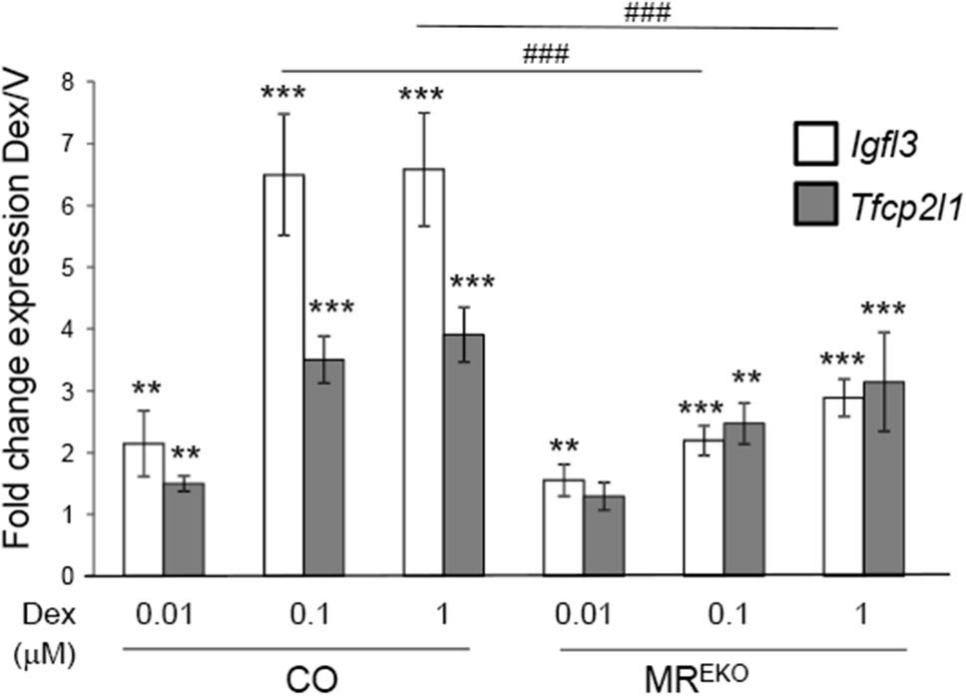
Glucocorticoid-regulation of target genes is paradoxically diminished in cultured MR^EKO^ *vs* CO keratinocytes. RT-qPCR analysis of gene expression in CO and MR^EKO^ keratinocytes treated with indicated concentrations of Dex for 3 h. Fold changes (Dex/Vehicle) in *Igfl3* and *Tfcp2l1* are shown. Statistically significant changes between Dex/V within each genotype are denoted by asterisks and significant differences between fold changes in CO and MR^EKO^ by pound signs (**, ^##^p < 0.01; ***, ^###^p < 0.001; n = 3).

These data were unexpected as we previously reported that the loss of MR function in keratinocytes, either in MR^EKO^ or in CO cells treated with the MR antagonist eplerenone, decreased Dex-mediated induction of a GRE-luc reporter^7^. Therefore, to evaluate this apparent paradox, we assessed *Igfl3* and *Tfcp2l1* expression in CO and MR^EKO^ keratinocytes treated with varied concentrations of Dex for 3 h (Fig. 5). Interestingly, at lower concentrations of Dex (10 nM), there was no difference in upregulation of *Igfl3* between genotypes; however the decreased induction in MR^EKO^ became clear at 100 nM. In the case of *Tfcp2l1*, there were no statistically significant differences at any concentration; however there was a trend towards decreased induction that was more evident at 100 nM.

**Figure 6:**
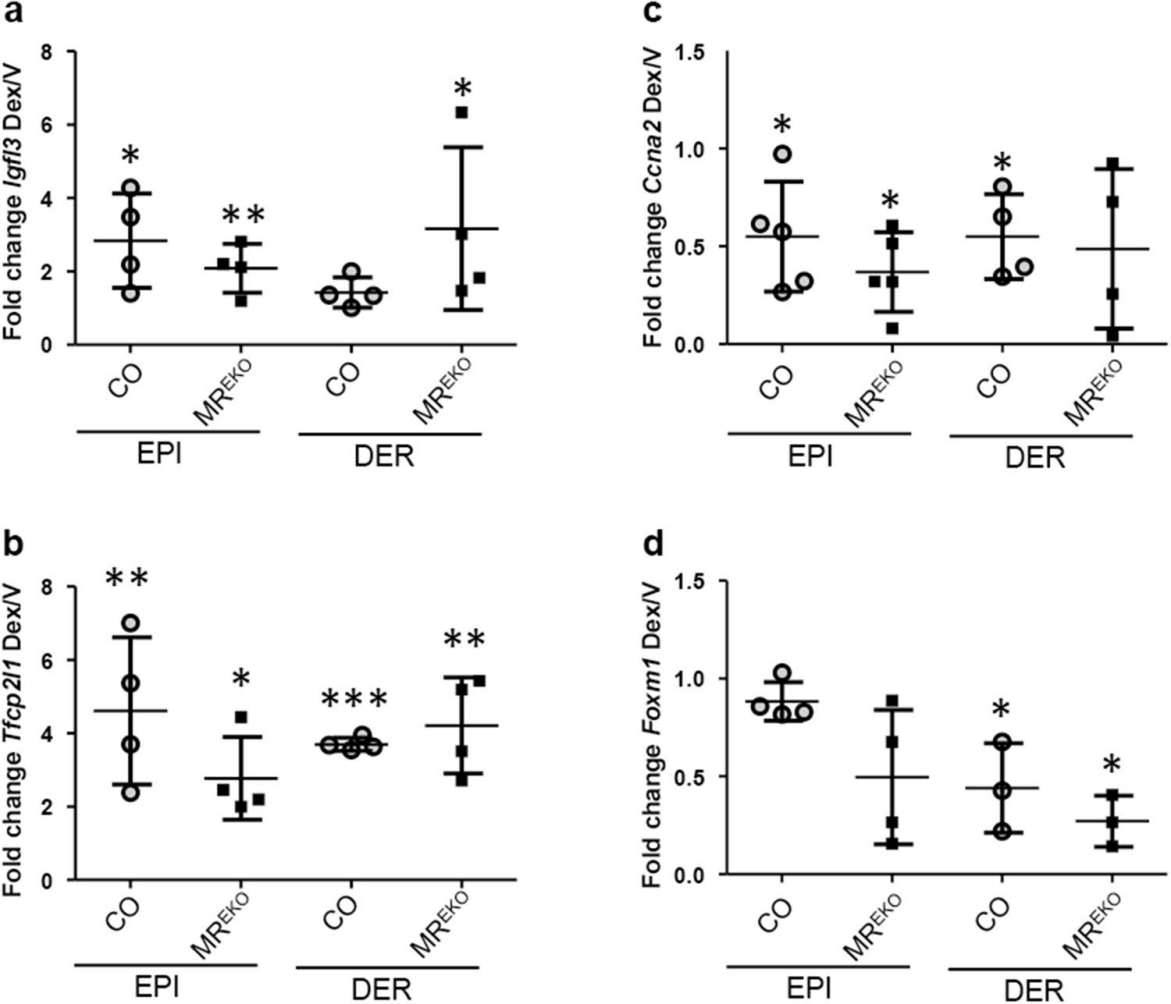
Evaluation of glucocorticoid-mediated gene regulation in CO and MR^EKO^ epidermis and dermis. Fold-change (FC) Dex/V expression of *Igfl3* (**a**), *Tfcp2l1* (**b**), *Ccna2* (**c**), *and Foxm1* (**d**), in epidermis and dermis of control (CO) and MR^EKO^ mice. Statistically significant changes between Dex/V within each genotype are denoted by asterisks (*p < 0.05; **p < 0.01; ***p < 0.001).

Next, we assessed the expression of several identified DEGs after Dex treatment in the epidermis and dermis of CO and MR^EKO^ mice (Fig. 6). Consistent with the results in cultured keratinocytes, *Igfl3* and *Tfcp2l1* showed a trend towards decreased induction in the epidermis of MR^EKO^ relative to CO (Fig. 6a,b). However, both genes showed a trend towards increased up-regulation in the dermis of MR^EKO^ *vs* CO (Fig. 6a,b). We also assessed the expression of genes repressed by Dex such as *Ccna2* and *Foxm1* (Fig. 4a). There was an overall trend towards strong repression of these genes in the epidermis and dermis of MR^EKO^ *vs* CO mice; however, there were no statistically significant differences among genotypes (Fig. 6c,d). These results suggest a shift in the homeostasis of MR^EKO^ skin compartments, altering GC responsiveness throughout the tissue.

Taken together our data show that epidermal GR and MR are required for appropriate response to topical GCs with their loss having repercussions on underlying dermis.

## Discussion

This study addresses for the first time the relative contribution of endogenous GR and MR to the transcriptional response to topically applied GCs. Our data showed that the epidermal loss of GR or MR resulted in opposite responses to GCs: while GR^EKO^ mice were refractory to Dex, MR^EKO^ featured both a higher number of Dex-regulated genes and an increased magnitude of response (Figs. 1, 3, and 6).

These data highlight the importance of the relative expression of both epidermal receptors for the GC response. While the loss of epidermal GR resulted in decreased GR activity in the dermal compartment (Fig. 2), the loss of epidermal MR had an impact on GC-dependent gene expression not only for keratinocytes but for the whole tissue (Fig. 4).

While previous data showed a central role for GR in the Dex response in keratinocytes^7,11,12^, this work demonstrates that epidermal GR is absolutely required for GC-dependent gene expression in the whole tissue. The lack of Dex responsiveness of GR^EKO^ skin also indicates that in the absence of GR, endogenous MR per se is not transcriptionally efficient. These data are consistent with the fact that in GR^EKO^ keratinocytes Dex response was restored upon transfection of GR or MR although the magnitude of the response was one order higher for GR relative to MR^7^. Also, in DKO cells, we showed that 40 fold more MR than GR was needed to achieve similar induction of a GRE-luc reporter^8^.

GC synthesis and release are under the control of the hypothalamic–pituitary–adrenal neuroendocrine axis, which acts as a major regulator of skin integrity and function. In addition to the adrenal source of GCs, the skin can produce steroidogenic enzymes and extra-adrenal GCs, allowing for an immediate tissue-specific response^21^. Local GC synthesis occurs in both epidermis and dermis, and plays a major role in cutaneous homeostasis and pathological conditions^22–27^. Indeed, it has been reported that local GC deficiency and reduced GR expression in human psoriatic lesions contributed to the pathogenesis of the disease^28,29^. Also, we reported that in homeostatic conditions, endogenous corticosterone levels in skin were significantly higher in GR^EKO^ relative to CO mice, likely as an attempt to compensate for the loss of GR in keratinocytes^28^. We speculate that the excess of locally produced corticosterone in GR^EKO^ mice is a major contributing factor to the dermal resistance to topically applied GCs.

Another possible mechanism for the GC resistance in the dermal compartment of GR^EKO^ is the constitutive up-regulation of p38 pathway (Fig. 2). While the increased p38 activity appears to be below a threshold for inducing overt changes in phenotype, it does leave the mice poised for increased susceptibility to inflammation^6,8,30^. It is also feasible that the main consequence of p38 activation in the dermis would be inactivation of dermal GR (Fig. 2). Given that p38 pathway is over activated in pathologies such severe asthma or COPD^12^, our data suggest that co-treatments of GCs and p38 antagonists may also be useful for treating skin inflammatory diseases.

Despite the high structural and functional homology between GR and MR, and in particular the identity in their DNA binding domain (greater than 90%), the global or epidermal-specific phenotypes of the knock-out mouse models for these transcription factors clearly indicate that they do not play overlapping functions^3^. It is thus expected that they regulate different gene subsets, as recently shown by in vitro and in vivo data^8,31–34^. It is also expected that the relative functions of GR and MR, either alone or combined depend on the cell-type. In this regard, the generation of double GR/MR tissue-specific knock-outs has demonstrated that while GR and MR cooperate to regulate skin morphogenesis^8^, these transcription factors play opposite roles in cardiomyocytes^35^.

The DEGs identified in in this study as well as the overrepresented processes are highly coincident with those recently reported in human skin treated with clobetasol^10^, further validating the use of mice to model skin responses to GCs. The fact that proliferation and apoptosis, two crucial processes modulated by GCs, were overrepresented in both CO and MR^EKO^ suggest that epidermal MR may not be strictly necessary for these GC actions. However, as proliferation and apoptosis were also overrepresented in MR^EKO^, it is feasible that the absence of epidermal MR modulates the sensitivity to GCs of specific gene subsets.

The idea that endogenous epidermal MR may modulate the skin sensitivity to GCs would support the topical use of MR antagonists in combination with GCs as an advantageous therapeutic approach. However, it is crucial to address first the question of whether an increased response to GCs is beneficial per se, or could rather mediate some of the GC-associated adverse effects; these options are not mutually exclusive^7^. It is known that the induction or repression of GC targets above or below proper threshold can contribute to detrimental effects of GC excess in several tissues. For example, up-regulation of *Zbtb16* and *Txnip*/*Vdup* has been reported in bone tissue in Cushing patients^36,37^. It is also worth noting the high overlap between the DEGs identified in this study and the gene expression changes in human skin of aged vs young subjects^10^.

The consequences of the higher response to topical GCs in MR^EKO^ vs CO skin are difficult to interpret. On one hand, the repression of *Foxm1*, a transcription factor contributing to the proliferation/differentiation balance in keratinocytes, as well as its target genes *Cenpf*, *Top2a*, *Ccna2*, *Birc5*, and *Plk1*, was twofold higher in MR^EKO^ vs CO (Dataset S1 and Fig. 4). Also, additional FOXM1 target genes such as *Ccnb1* and *Ccnb2* were down-regulated by Dex only in MR^EKO^ skin (Dataset S1). However, several anti-inflammatory genes such as *Tsc22d3* or the GC-responsive chemokine *Cxcl10* were similarly regulated by Dex in both genotypes (Fig. 4). While these data indicate that GCs differentially affect specific gene subsets depending on the presence or absence of epidermal MR, it is difficult to conclude whether combined treatments with MR antagonists and GCs would be superior to GCs alone.

Recent work has demonstrated that GR can form diverse oligomerization complexes including tetramers formed by dimers of dimers^38^. Whether GR and MR can also form higher-order complexes, including tetramers, cannot be excluded. Also, other mechanisms such as the tethering of the MR upon GR-bound DNA have been reported to increase GC transcriptional responses, independently of the MR DBD^39^.

We speculate that the lack of epidermal MR could alter the GR recruitment to the regulatory sequences of GC-target genes or modify the pattern of GR interaction with co-regulators and/or chromatin modifying proteins. While these hypotheses need to be confirmed by high throughput approaches, the identification of the transcriptomic profiles elicited by topical GCs in this work contributes to understanding the relative roles of the endogenous epidermal GR/MR in transcriptional regulation in the skin.

## Materials and methods

### Mice

Mouse experimentation was performed following current Spanish and European regulations and subjected to approval by the ethics committee of the Instituto de Biomedicina de Valencia IBV-CSIC (ID for project SAF2014-59474-R; SAF2017-88046-R). Groups of 3 to 6 of mice were housed in conventional cages with ad libitum access to food and water, controlled temperature (20–22 °C), humidity (50–60%) and a 12-h light:dark cycle.

Mice with the third exon of *Nr3c1*^40^ and*/*or *Nr3c2*^41^ flanked by loxP sites were crossed with those expressing Cre under the control of the keratin 5 promoter (K5-Cre)^42^ to generate mice lacking epidermal GR (GR^EKO^)^6^, MR (MR^EKO^)^7^, or both (DKO)^8^. Mice were kept on the B6D2 background and littermates with loxP flanked *Nr3c1* and/or *Nr3c2* were used as controls. Genotyping was performed using primers in Table S1.

Dorsal flanks of 2-month old female mice in the telogen phase of the hair cycle were shaved 48 h prior to topical treatment with Vehicle (acetone; left flank) or 8 µg Dex (right flank; Sigma), which was carried out in the morning. Upon sacrifice at indicated timepoints, skin was collected and snap frozen in liquid nitrogen.

### RNA isolation and quantitative RT-PCR

RNA from mouse cells or tissues was isolated using Trizol (Invitrogen, Carlsbad, California, USA). Tissues were homogenized in Trizol with a polytron (PT1600E, Kinematica, Luzern, Switzerland). The RevertAid H Minus Reverse Transcriptase kit (ThermoFisher) was used to generate cDNA. The Quant Studio 5 Real-Time PCR System (Applied Biosystems, Foster City, California, USA) was used for QPCR with gene specific oligonucleotides (0.3 μM each) and the FastStart Universal SYBR Green Master mix ROX (Merck). Cts were normalized to those of the housekeeping gene *Hprt1*. Technical triplicates were used; and 3–6 biological replicates per experimental group were assessed to calculate the mean value ± SD. Primer sequences are in Table S1 and those from Primer Bank are indicated in table with corresponding ID^43^.

### RNA-sequencing and bioinformatic analysis

CO, GR^EKO^, MR^EKO^ or DKO mice (n = 3/genotype) were treated with vehicle or 8 µg Dex for 24 h and then sacrificed and RNA was prepared from dorsal skin. Integrity of total RNA in the samples was assessed with the Agilent 2100 Bioanalyzer using the RNA 6000 nano Kit (Agilent Technologies, Santa Clara, CA, USA). Purified RNA from each sample was prepared for sequencing using the TruSeq Stranded mRNA Sample Preparation Kit with PolyA selection for ribo depletion (Illumina Inc., San Diego, CA, USA). Libraries were sequenced on Illumina NextSeq 500 instrument. Depth of 2237 million single 75 pb reads were generated for each sample.

Raw sequence reads were checked for quality, adapter trimmed and filtered using Cutadapt v1.8.3^44^ and FastQC v0.11.8^45^. Trimmed sequences were mapped to the *Mus musculus* genome (assembly: GRCm38, annotation: Ensembl release 87) using TopHat2 (v2.1.0)^46^. Seqmonk version 1.41^47^ was used for quality control, visualization, and quantification. Raw read counts were generated by counting uniquely mapped reads over protein-coding genes using the RNA-seq quantification pipeline assuming opposing strand specificity. Downstream expression analysis was conducted with the EdgeR R/Bioconductor package (v3.20.9) using R (v3.4.4)^48^. Genes that not satisfy the condition of having at least 1 count-per-million in at least 3 samples were removed. Differential gene expression analyses between experimental defined groups were performed using generalized linear model approach and quasi-likelihood F-test 40. Benjamini–Hochberg false discovery rate (FDR) q values ≤ 0.05 was considered to be significant. The heat map was generated from a cluster analysis of the normalized read counts (scaling in a per gene basis using the z-score ((x − µ)/σ), euclidean distance, complete-linkage) using the 'pheatmap' package (v 1.0.12) in the R statistical language (v 3.4; https://CRAN.R-project.org/package=pheatmap). Gene ontology was determined using DAVID functional clustering with an EASE score less than 0.05 and medium stringency^48,49^.

### Separation of skin compartments

Following excision of dorsal skin, the hypodermis was removed by scraping the underside of the skin with a scalpel. Next, the remaining skin was floated dermal side down in 3.8% ammonium thiocyanate in DPBS for 10 min at room temperature. The epidermis was then separated from the dermis; both tissues were washed extensively in DPBS, minced and snap frozen in liquid N_2_.

### Immunoblotting

Lysates from whole skin, epidermis, dermis or cultured cells were prepared as previously described^7^ using buffers supplemented with Complete protease and phosphatase inhibitor cocktails (Merck). Protein concentrations were measured using Bradford reagent (BioRad, Hercules, California, USA) and 20–30 µg of protein/sample was boiled in Laemmli buffer, separated on SDS-PAGE, and transferred to Hybond ECL nitrocellulose (GE Healthcare, Illinois, USA). Nitrocellulose membranes were stained with Ponceau S (Merck) to verify equal protein loading and transfer prior to blocking and antibody incubations. Primary antibodies used were from Cell Signalling Technology (Danvers, Massachusetts, USA): p-GR Ser^211^, #4161S, 1/2000; p-ERK Thr^202^/Tyr^204^, #4376, 1/1000; p-p38 Thr^180^/Tyr^182^ #4631, 1/1000; p-JNK Thr^183/^Tyr^185^ #9251, 1/1000, and JNK #9252, 1/1000; Santa Cruz Biotechnology (Dallas, Texas, USA): GR, sc1004, 1/2000; Sigma: actin, A2066, 1/4000; and tubulin, T6199, 1/4000. Peroxidase-conjugated secondary antibodies were from GE Healthcare: anti-rabbit, NA934 and anti-mouse NXA931. Immunoreactive bands were detected using Pierce ECL Plus Western Blotting Substrate (ThermoFisher) and the ImageQuant 4000 Biomolecular Imager (GE Healthcare). Band intensities were quantitated using Image J software and were normalized to the loading controls, actin, or tubulin.

### Cell culture

Unless otherwise mentioned cell culture reagents were from Biowest (Nuaillé, France).

J2-3T3 fibroblasts were maintained in DMEM supplemented with 7.5% bovine serum (Gibco, ThermoFisher), 100 U/ml penicillin/100 μg/ml streptomycin, 2 mM glutamine and 0.25 μg/ml amphotericin B.

Fibroblasts were used only as feeders for culture and maintenance of the spontaneously immortalized keratinocyte cell lines, which was previously described^7,8,11^. For that purpose, mitotically inactivated J2-3T3 fibroblasts (treated with mitomycin C (Panreac, Castellar del Vallès, Spain) were plated in flasks coated with type I collagen from rat tail (Gibco, ThermoFisher). Next, keratinocytes were added to flasks and cells were cultured in keratinocyte medium: calcium-free DMEM (Gibco, ThermoFisher) and Ham’s F12 mixed at a 3:1 ratio and supplemented with 1.8 × 10 − 4 mol/l adenine (Sigma), 0.35 mM calcium (Sigma), 7.5% FBS Gold, 100 U/ml penicillin/100 μg/ml streptomycin, 2 mM glutamine, 0.25 μg/ml amphotericin B, 5 μg/ml insulin (Sigma), 10^−10^ M cholera toxin (Sigma), and 10 ng/ml EGF (Peprotech, London, UK).

For experimentation, fibroblasts were removed from flasks with Versene and keratinocytes were plated and cultured alone in collagen I-coated tissue culture dishes in keratinocyte medium until around 80% confluent. At this point, cells were washed with DPBS and cells were cultured for 24 h in keratinocyte medium containing charcoal-stripped FBS. Then, keratinocytes were treated with the indicated concentrations of Dex for 3 h.

### Statistical analysis

Experimental data were analyzed with Microsoft Excel and IBM SPSS Statistics 25 software. In all graphs mean values ± SD are shown. When statistical analysis was performed with relative values, data were first subjected to logarithmic transformation. The Levene’s test was used to determine whether samples within groups had equal variance before parametric testing. The Student’s unpaired two-tailed t-test was used for comparisons between two experimental groups. For comparisons among higher numbers of experimental groups, the one-way ANOVA and post hoc Tukey multiple comparison test was used.

## Supplementary information

Supplementary Information 1.

Supplementary Dataset 1.

Supplementary Dataset 2.

## Data Availability

All data generated or analyzed during this study are included in this published article (and its Supplementary Information files).
