# Supplementary material for: Glucocorticoid-dependent transcription in skin requires epidermal expression of the glucocorticoid receptor and is modulated by the mineralocorticoid receptor
Source: Sci Rep. 2020 Nov 3;10:18954. doi: 10.1038/s41598-020-75853-5 (PMC7609727; doi:10.1038/s41598-020-75853-5)

Glucocorticoid-dependent transcription in skin requires epidermal expression of the glucocorticoid receptor and is modulated by the mineralocorticoid receptor

Lisa M. Sevilla[1], Judit Bigas[1], Álvaro Chiner-Oms[1], Iñaki Comas[1], Vicente Sentandreu[2], and Paloma Pérez[1, #]

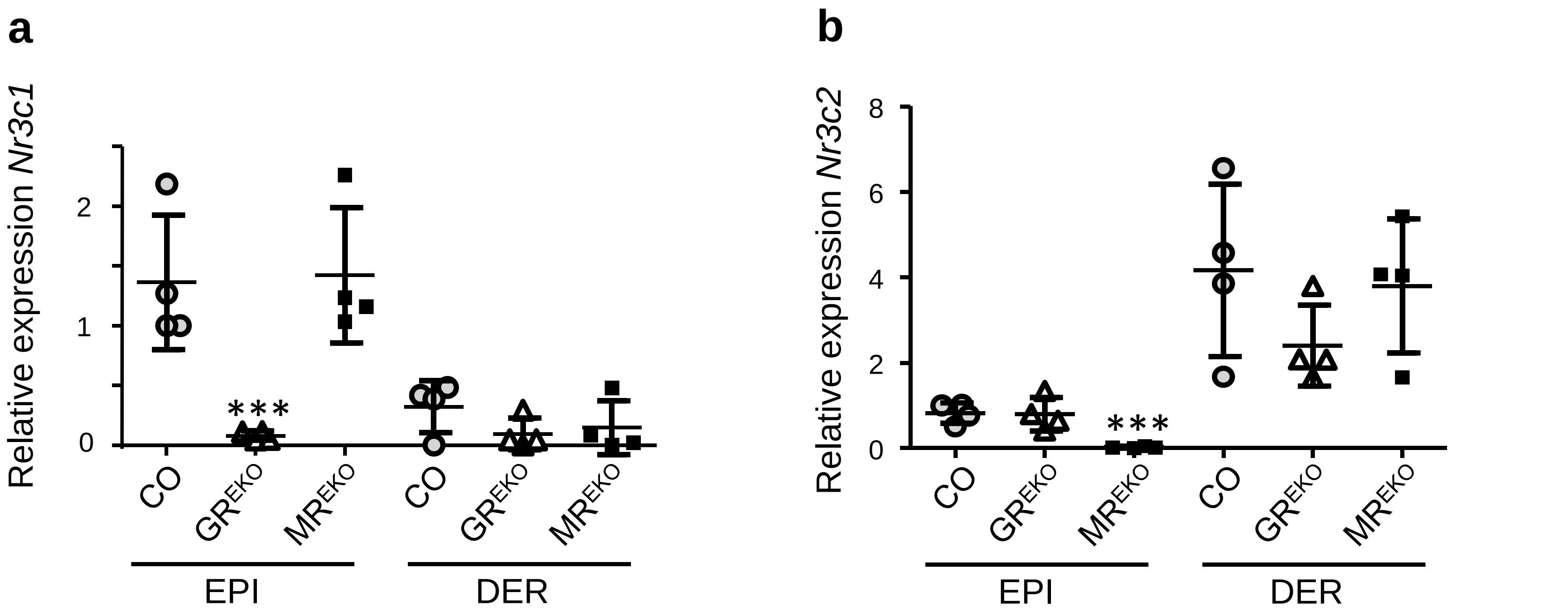

Figure S1. **Loss of epidermal GR or MR does not affect expression of epidermal MR or GR, respectively; or that of either receptor in dermis.**
Relative basal expression of (a) *Nr3c1* (GR) or (b) *Nr3c2* (MR) in epidermis (EPI) and dermis (DER) of vehicle-treated CO, GR^{EKO}, and MR^{EKO} mice, was assessed by RT-qPCR. Asterisks indicate statistically significant differences relative to CO (***p<0.05).

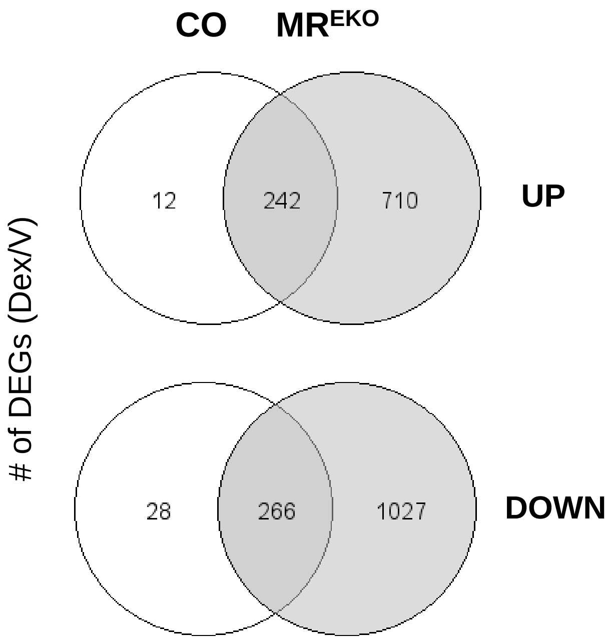

Figure S2. **Venn diagrams showing the number (#) of common and unique Differentially Expressed Genes (DEGs) in CO and MR^{EKO} skin following topical treatment with Dex *vs* vehicle (V) for 24h.**
Up- and down-regulated genes are shown separately. Note the high overlap of CO DEGs with those in MR^{EKO} skin.

Sevilla_Table S1. Primers used for RT-QPCR and genotyping

RT-QPCR primers

**Gene**	**Forward**	**Reverse**	**Product size (bp)**	**Primers from Primerbank ID**
Ccl20	GCCTCTCGTACATACAGACGC	CCAGTTCTGCTTTGGATCAGC	146	Primer Bank ID8394248a1
Ccna2	CAACCCCGAAAAACTGGCGC	AAGAGGAGCAACCCGTCGAG	118	
Ccnd1	CATCAAGTGTGACCCGGACTG	CCTCCTCCTCAGTGGCCTTG	116	
Cxcl10	CCAAGTGCTGCCGTCATTTTC	GGCTCGCAGGGATGATTTCAA	157	Primer Bank ID10946576a1
Cxcl9	TCCTCTTGGGCATCATCTTCC	TTTGTAGTGGATCGTGCCTCG	110	Primer Bank ID6678880a1
Ddit4	GGGCCGGAGGAAGACTCCTCATA	CTGTATGCCAGGCGCAGGAGTTC	218	
Fkbp51	CTTGGACCACGCTATGGTTT	AACGACTCTGAGGCTTTGGA	290	
Hprt1	TCAGTCAACGGGGGACATAAA	GGGGCTGTACTGCTTAACCAG	142	Primer Bank ID7305155a1
Igfl3	GTGGTGACCAGCCTTGAGAG	GCACCCCAATGCTTCAGGTA	94	
Il1b	GCAACTGTTCCTGAACTCAACT	ATCTTTTGGGGTCCGTCAACT	89	
Mt1	CTCCGTAGCTCCAGCTTCAC	AGGAGCAGCAGCTCTTCTTG	137	
Nr3c1	TGCTATGCTTTGCTCCTGATCTG	TGTCAGTTGATAAAACCGCTGCC	299	
Nr3c2	GTGGACAGTCCTTTCACTACCG	TGACACCCAGAAGCCTCATCTC	286	
S100a8	GGAGTTCCTTGCGATGGTGAT	TCTGCTACTCCTTGTGGCTGTCT	68	
Sprr2d	TGGTACTCAAGGCCGAGA	TTTGTCCTGATGACTGCTGAAGAC	336	
Tfcp2l1	AGGTGCTGACCTCCTGAAGA	GTTTTGCTCCAGCTCCTGAC	145	
Tsc22d3 (Gilz2)	CTGTTGGCCTCGACTGCTG	GCCGAAAGTTGCTCACGAAG	111	Primer Bank ID11907580a1

Genotyping primers

**Mouse line**	Primer name	
Mrflox, MREKO,Dflox, DKO	MR_F	CTGGAGATCTGAACTCCAGGCT
	MR_RA	TAGAAACACTTCGTAAAGTAGAGCT
	MR_RB	CCTAGAGTTCCTGAGCTGCTGA

**Product size WT**	**Product size MRflox**	**Product size MREKO**
285	335	390

GRflox, GREKO, Dflox, DKO	GR1	GGCATGCACAATTACGGCCTTCT
	GR4	GTGTAGCAGCCAGCTTACAGGA
	GR8	CCTTCTCATTCCATGTCAGCATGT

**Product size WT**	**Product size GRflox**	**Product size GREKO**
225	275	390

Molecular weight marker: Thermo Scientific Spectra multicolor broad range protein ladder.

Fig 2b

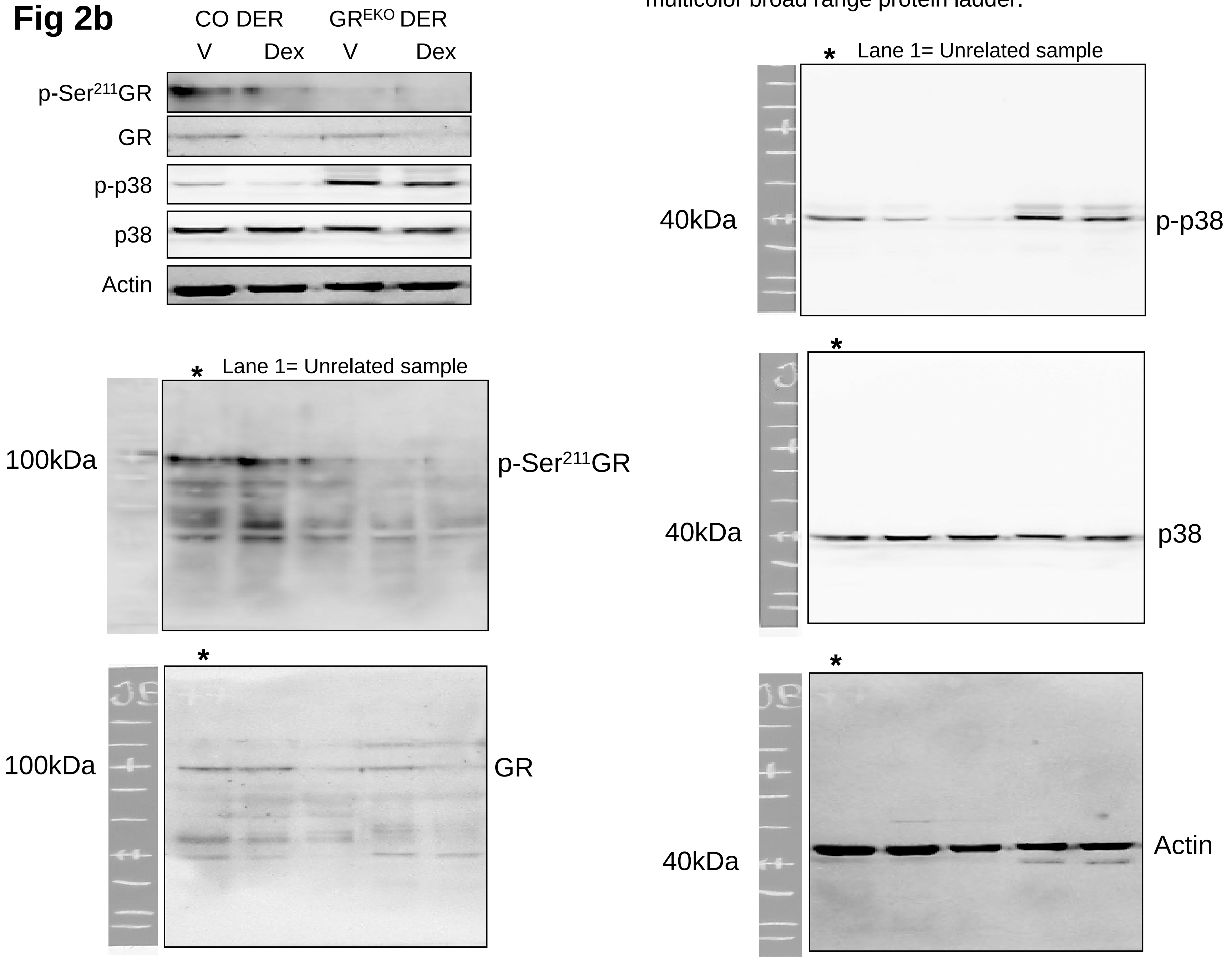

Supplement: Supplementary file 1 — Supplementary Information 1. [file 41598_2020_75853_MOESM1_ESM.pdf]
